# Effects of gut microbial therapy on lipid profile in individuals with non-alcoholic fatty liver disease: an umbrella meta-analysis study

**DOI:** 10.1186/s13643-023-02299-x

**Published:** 2023-08-21

**Authors:** Amirhossein Naghipour, Ehsan Amini-Salehi, Mahdi Orang Gorabzarmakhi, Milad Shahdkar, Bahman Fouladi, Iraj Alipourfard, Zahra Momayez Sanat

**Affiliations:** 1 Department of Pharmaceutics, Faculty of Pharmacy, Kerman University of Medical Sciences, Kerman, Iran; 2 Gastrointestinal and Liver Diseases Research Center, Guilan University of Medical Sciences, Rasht, Iran; 3 Student Research Committee, School of Medicine, Guilan University of Medical Sciences, Rasht, Iran; 4 Guilan University of Medical Sciences, Rasht, Iran; 5 Pediatric Gastroenterology and Hepatoloy Research center, Zabol University of Medical Sciences, Zabol, Iran; 6 Department of Parasitology and Mycology, School of Medicine, Zabol University of Medical Sciences, Zabol, Iran; 7 Institute of Physical Chemistry, Polish Academy of Sciences, Marsaw, Poland; 8 Digestive Disease Research Institute, Tehran University of Medical Sciences, Tehran, Iran

**Keywords:** Non-alcoholic fatty liver disease, Probiotics, Microbial therapy, Lipid profile, Umbrella meta-analysis, Systematic review

## Abstract

**Background:**

Non-alcoholic fatty liver disease (NAFLD), the most common liver disease, is closely associated with metabolic conditions such as obesity and diabetes mellitus, which significantly impact human health outcomes. The impaired lipid profiles observed in NAFLD individuals can further contribute to cardiovascular events. Despite the high prevalence of NAFLD, there is currently no confirmed intervention approved for its treatment. This study aimed to summarize the results of meta-analysis studies of randomized control trials assessing the impact of gut microbial therapy (probiotics, synbiotics, and prebiotics) on the lipid profile of individuals with NAFLD.

**Methods:**

A systematic search was conducted on PubMed, Scopus, Web of Science, and Cochrane Library up to November 1, 2022. Meta-analyses surveying the impact of microbial therapy on lipid profile parameters (triglyceride (TG), high-density lipoprotein (HDL), low-density lipoprotein (LDL), and total cholesterol (TC)) in the NAFLD population were included in our umbrella review. The final effect size (ES) was estimated, and sensitivity and subgroup analyses were performed to explore heterogeneity.

**Results:**

Fifteen studies were included in this umbrella review. Microbial therapy significantly reduced TG (ES − 0.31, 95% CI − 0.51, − 0.11, *P* < 0.01), TC (ES − 1.04, 95% CI − 1.46, − 0.61, *P* < 0.01), and LDL (ES − 0.77, 95% CI − 1.15, − 0.39, *P* < 0.01) in individuals with NAFLD. However, the effect on HDL was not statistically significant (ES − 0.06; 95% CI − 0.19, 0.07, *P* = 0.39).

**Conclusion:**

Considering the absence of approved treatments for NAFLD and the promising role of microbial therapies in improving the three lipid profiles components in individuals with NAFLD, the use of these agents as alternative treatment options could be recommended. The findings underscore the potential of gut microbial therapy, including probiotics, synbiotics, and prebiotics, in managing NAFLD and its associated metabolic complications.

**Trial registration:**

PROSPERO (CRD42022346998).

**Graphical Abstract:**

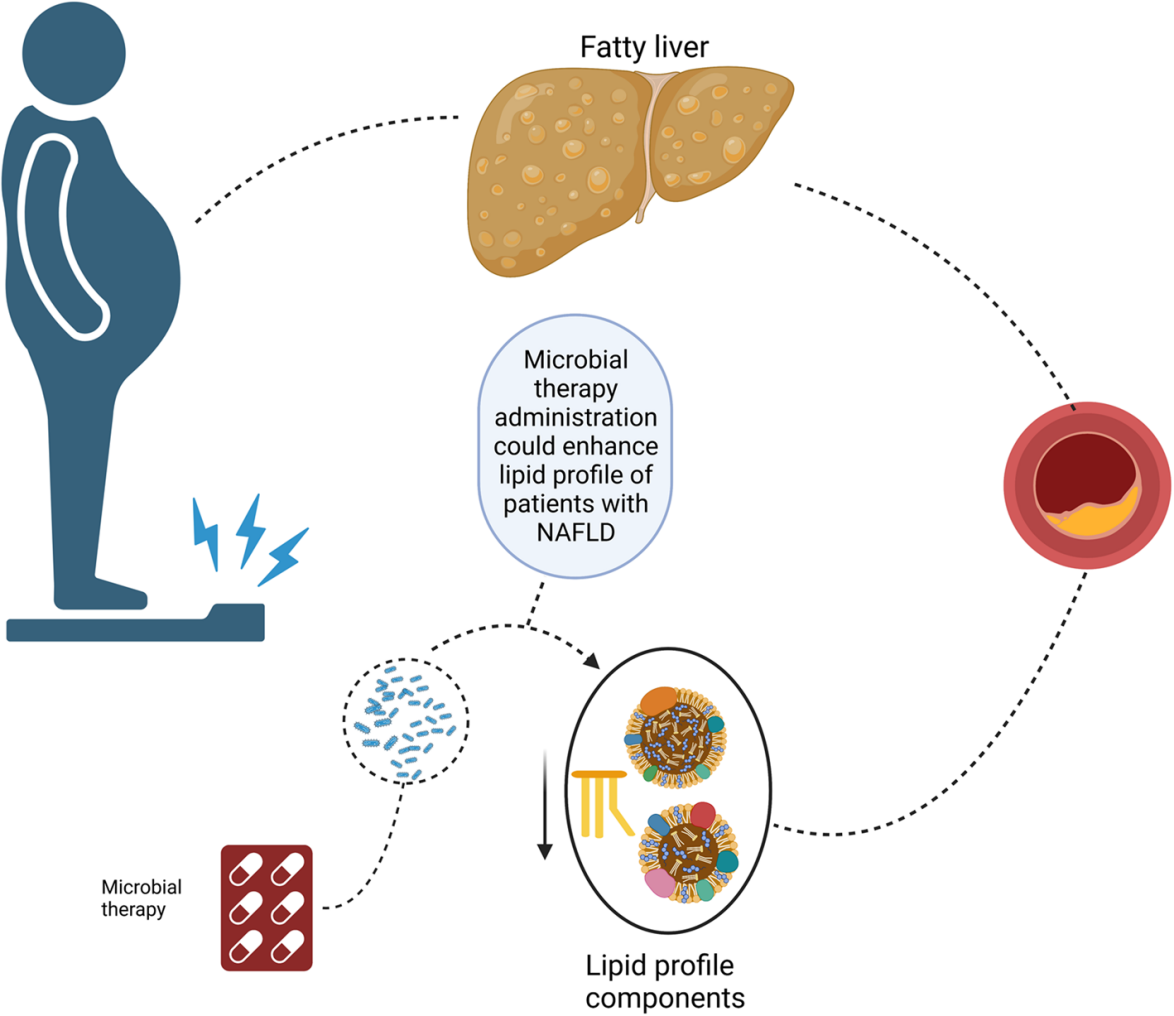

**Supplementary Information:**

The online version contains supplementary material available at 10.1186/s13643-023-02299-x.

## Background

Non-alcoholic fatty liver disease (NAFLD) is identified by excessive fat accumulation in the liver, which consequently promotes necroinflammation and fibrosis, ultimately leading to liver failure [1–4]. This disease includes various conditions, from simple steatosis to hepatic cirrhosis [5–8]. The prevalence trend of NAFLD showed an increase of 0.7% annually, and the global prevalence of NAFLD is estimated at 29.8%. Although NAFLD is highly prevalent on all continents, South America and North America were reported as having the highest rates of NAFLD, with a prevalence of 35.7% and 35.3%, respectively [9]. NAFLD is considered the most common cause of chronic liver disease [10].

Although the pathogenesis of NAFLD is not fully understood, nutritional, environmental, and genetic factors modifying lipid and glucose metabolism are involved in the development of this condition [11–13]. Among the plethora of risk factors, recent evidence has pointed out the role of gut dysbiosis and its metabolites in the pathophysiology of NAFLD [14]. Recent investigations suggest intestinal dysbiosis can affect gut permeability, the innate immune system, the fermentation of indigestible carbohydrates, and the intestinal production of short-chain fatty acids, which can lead to NAFLD [15, 16]. In addition, evidence shows differences between the gut microbiota of healthy subjects and those with NAFLD and that the importance of diet in NAFLD is partly due to its ability to change the gut microbiome [11].

NAFLD is related to other diseases like diabetes mellitus, obesity, metabolic syndrome, hypertension, renal disorders, and cardiovascular diseases [17–23]. In addition to the relationship between NAFLD and other health conditions, this disease caused a significant burden globally [24–26]. The current known pharmacological treatments for NAFLD are few, and the primary focus for NAFLD management is on lifestyle modification, including weight loss, physical activity, and diet regimen [14, 27]. Although there is no specific treatment for NAFLD, it is hoped microbial therapies, including probiotics, prebiotics, and synbiotics, will provide a new therapeutic method for the treatment by manipulating intestinal microbiota [28, 29]. Probiotics are defined as live microorganisms in the diet which can regulate gut microbiota and are helpful for individuals' health [30]. Prebiotics are indigestible foods that can selectively provoke some bacterial production or activity in the human body [31], and synbiotics are a combination of both probiotics and prebiotics [32].

Previous investigations showed the promising effects of microbial therapies on NAFLD; however, the results were controversial, and up to now, no medications have been approved for the treatment of NAFLD patients [14, 33–37]. Hence, we aimed to conduct an umbrella review of meta-analysis studies to provide comprehensive, evidence-based information on microbial therapy’s effects on the NAFLD population’s lipid profile.

## Methods

We conducted this umbrella review (a systematic review on different meta-analyses) based on the Cochrane Handbook for Systematic Reviews of Interventions [38]. The reporting of the results was based on Preferred Reporting Items for Systematic Reviews and Meta-Analyses (PRISMA) guidelines [39].

### Search strategy and study selection

Four international databases, including PubMed, Web of Science, Scopus, and Cochrane Library, were searched from inception until November 1, 2022. To increase the quality of searching, we consulted information specialists and manually searched the reference list of relevant studies. No language restriction was admired. We used EndNote X20 for managing the searched studies. The search strategy and keywords are provided in Table S 1.

### Inclusion and exclusion criteria

Meta-analyses of randomized control trial (RCT) studies surveying the effect of probiotics, prebiotics, and synbiotics on the lipid profile **(**triglyceride (TG), high-density lipoprotein (HDL), low-density lipoprotein (LDL), and total cholesterol (TC)) of the NAFLD population were eligible for our umbrella review. Systematic reviews without meta-analysis, narrative reviews, letters to the editor, network meta-analyses, and original studies were excluded. Two reviewers selected the studies based on the inclusion criteria.

### Quality assessment

Two reviewers assessed the quality of included meta-analyses using the AMSTAR 2 checklist, and any disagreements were resolved by a third researcher. AMSTAR 2 consists of 16 questions with the answers “yes,” “no,” or “partial yes.” The final assessment is qualitatively reported as “high,” “moderate,” “low,” or “critically low” based on the answers of reviewers [40]. The quality assessment of included studies is provided in Table S 2.

### Data extraction

Two reviewers independently extracted data from the included studies, and the third researcher resolved disagreements. The following data were extracted from each study: name of the first author, country of study, protocol registry number, source of funding, searched engines, number of included studies, methods for assessing the source of heterogeneity and publication bias, and effect size (ES) and confidential interval (CI) of HDL, LDL, TG, and TC. The extracted data were entered into a predesigned Excel sheet. We contacted the studies corresponding for any missing data.

### Data synthesis

ES and CI of the included meta-analyses were obtained to determine the overall effect. We assessed the between-study heterogeneity using *I*^*2*^ statistics and Cochrane’s *Q* test. High heterogeneity was considered when *I*^2^ > 50% and *P* value < 0.1. We used the random effect model when heterogeneity existed; otherwise, fixed effect model was applied. To assess the source of heterogeneity, we conducted subgroup analysis based on the total sample size of the meta-analyses, quality of meta-analyses, country, type of reporting units, type of intervention, availability of previous protocol, and source of funding. We also performed sensitivity analysis to assess the effect of every single study on the overall effect. The publication bias was assessed by visual inspection of the funnel plot and Egger regression test, and *P* value < 0.1 was determined as the level of significance [41, 42]. For any suspected asymmetry in the funnel plot, “trim and fill” analysis was conducted.

## Results

**Fig. 1:**
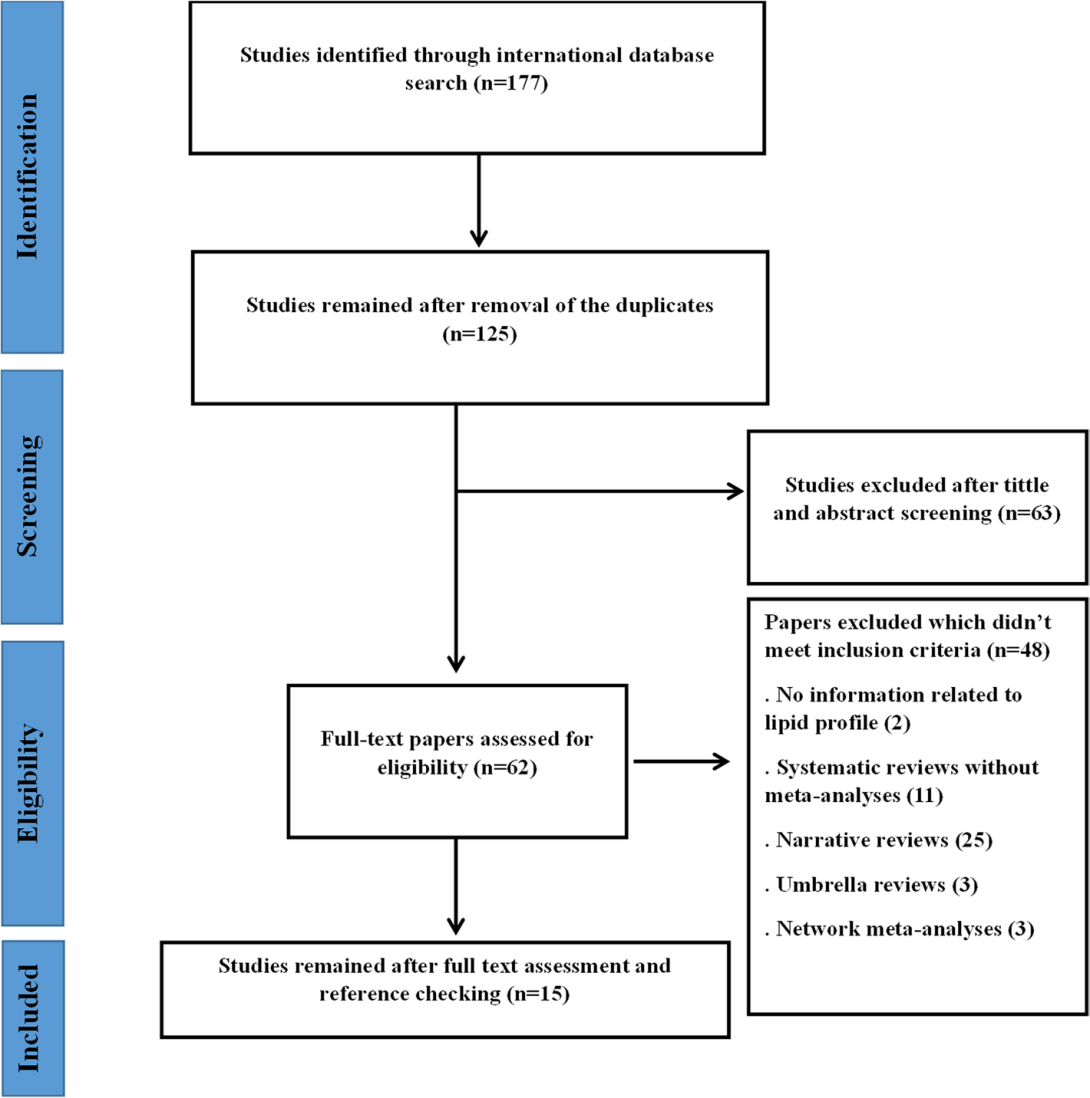
Study selection process

A total number of 177 studies were identified after searching the electronic databases. Among the search studies, 52 articles were duplicates, and the remaining went for the title and abstract screening. From the 125 studies, 63 studies got excluded, and 62 articles went for full-text assessment. Based on the inclusion criteria, 14 studies were selected for the analysis. Moreover, one study was found through reference search. A total number of 15 studies went for the final analysis. Figure 1 illustrates the study selection process.

### Studies characteristics

Among the included studies, eight were from China; three were from the USA; one was from Iran, one was from Greece, one was from India, and one was from France. Duration of intervention ranged from 2 to 28 weeks in the original studies within meta-analyses. The number of included original studies within meta-analyses varied from 4 to 29, and the sample sizes ranged from 134 to 2110. Four studies registered a previous protocol for their meta-analysis in International Prospective Register of Systematic Reviews **(**PROSPERO), and one study was registered in Open Science Framework (OSF). Eight studies were funded, four did not have any funding source, and the rest did not determine their funding support in their article. Eight studies assessed probiotics as their intervention, four assessed probiotics and synbiotics, one assessed synbiotics, and two assessed probiotics, prebiotics, and synbiotics. TC, TG, HDL, and LDL were evaluated in 11, 13, 9, and 9 studies, respectively. Detailed information about the characteristics of included studies is presented in Table 1.

**Table 1:** Characteristics of included studies

First author/year of publication	Protocol registry	Outcomes	Funding	Risk of bias assessment tool	Number of included studies/total sample size	Data bases/ date of search	Source of heterogeneity assessment methods	Publication bias assessment method	Country	Intervention/duration of treatment
GKIOURTZIS, 2022 [43]	(OSF) https://osf.io/qbw9h	TC, TG	Not reported	Cochrane	4/238	MEDLINE/PubMed, Scopus and Embase/ September, 2021	No assessment	Funnel plots, trim-and-fill, and Egger’s test	Greece	Probiotics (8 to 16 weeks)
HUANG, 2022 [44]	No previous protocol registry	TG, HDL, LDL	Funded	Cochrane and Newcastle–Ottawa Scale	24/1403	Embase, PubMed, and Web of Science/ January, 2011, to December, 2021	Sensitivity analysis, subgroup analysis	Begg's test	China	Probiotics (4 to 24 weeks)
LI, 2022 [45]	(PROSPERO) CRD42021288543	TC, TG, HDL, LDL	Funded	Cochrane	29/2110	PubMed, Embase, the Cochrane Library, Clinical trails.gov, and China National Knowledge Infrastructure/ January, 2000 to September, 2021	Sensitivity analysis, subgroup analysis	Visual inspection of funnel plots, Egger test, Trim and fill analyses	China	Probiotics, prebiotics, synbiotics (8 to 24 weeks)
YANG, 2021 [46]	No previous protocol registry	TC	No funding	Cochrane, Jadad	9/352	PubMed, Cochrane, MEDLINE, Web of Science and Embase/April, 2021	Sensitivity analysis, subgroup analysis	No assessment	China	Probiotics (8 to 48 weeks)
KOUTNIKOVA, 2019 [47]	(PROSPERO) CRD42016033273	TG	Funded	PEDro	12/660	PubMed/MEDLINE, EMBASE and the Cochrane Central/ 1990 to June 2018	Sensitivity analysis, subgroup analysis	Funnel plots or simple scatterplots, Egger’s test and Begg’s rank correlation test, trim, and fill	France	Probiotics (2 to 28 weeks)
XIO, 2019 [48]	No previous protocol registry	TC, TG, HDL, LDL	Funded	Cochrane, Jadad	28/1555	PubMed, Embase, Cochrane Library, Web of Science, OVID, China National Knowledge Infrastructure, VIP Database for Chinese Technical Periodicals, China Biology Medicine disc, and Wan fang Database/April, 2019	Meta-regression, sensitivity analysis, subgroup analysis	Visual inspection of Funnel Plots, Egger’s Test, trim, and fill method	China	Probiotics (4 to 28 weeks)
LIU, 2019 [49]	No previous protocol registry	TC, TG, HDL, LDL	Not reported	Cochrane	15/782	PubMed, Cochrane, and Embase/April, 2019	Sensitivity analysis, subgroup analysis	No assessment	China	Probiotics and synbiotics (8 to 28 weeks)
KHAN, 2019 [50]	No previous protocol registry	TC, TG, HDL, LDL	No funding	Cochrane	12/782	PubMed/MEDLINE, and Google Scholar/June,2018	Sensitivity analysis	No assessment	USA	Probiotics and synbiotics (8 to 24 weeks)
SHARPTON, 2019 [51]	(PROSPERO) CRD42018091455	TG	Funded	Cochrane	21/1252	PubMed/MEDLINE, Embase, and the Cochrane Library/ January, 2005 to December, 2018	Subgroup analysis, sensitive analysis and meta regression	Begg’s rank correlation test, and Egger’s regression test	USA	Probiotics and synbiotics (8 to 28 weeks)
HADI, 2019 [36]	No previous protocol registry	TC, TG, HDL, LDL	No funding	Jadad	11/419	PubMed, Scopus, ISI Web of science and Google Scholar/December, 2017	Sensitivity analysis, subgroup analysis	Begg’s rank correlation test, and Egger’s regression asymmetry test	Iran	Synbiotics (8 to 28 weeks)
TANG, 2019 [52]	(PROSPERO) CRD42019128193	TC, TG, HDL, LDL	Funded	Cochrane	22/1356	PubMed, Embase, the Cochrane Library, the Web of Science; China National Knowledge Infrastructure (CNKI), Wan Fang Data, and VIP/ April, 2019	Sensitivity analysis, Subgroup analysis	Egger’s test	China	Probiotics (4 to 24 weeks)
LOMAN, 2018 [53]	No previous protocol registry	TC, TG, HDL, LDL	Funded	Cochrane	25/1309	PubMed and Embase/December, 2017	Subgroup analysis	Visual inspection of the funnel plot and Begg’s and Egger’s tests	USA	Probiotics, prebiotics, synbiotics (8 to 24 weeks)
LAVEKAR, 2017 [54]	No previous protocol registry	TG	No funding	Jadad	7/296	PubMed, Cochrane, Embase, February, 2016	Sensitivity analysis	No assessment	India	Probiotics (8 to 28 weeks)
GAO, 2016 [55]	No previous protocol registry	TC, TG, HDL, LDL	Funded	Cochrane	9/535	Cochrane Library, PubMed/MEDLINE, EBSCO, OVID, SCI, CNKI, and VIP/July, 2015	Sensitivity analysis, subgroup analysis	No assessment	China	Probiotics (4 to 24 weeks)
MA, 2013 [56]	No previous protocol registry	TC, HDL, LDL	Not reported	Jadad	4/134	Medline, Embase, Web of Science, Chinese Biomedicine Database and the China Journal Full Text/Not reported	No assessment	No assessment	China	Probiotics (8 to 24 weeks)

### Effects of microbial therapy on TG

**Fig. 2:**
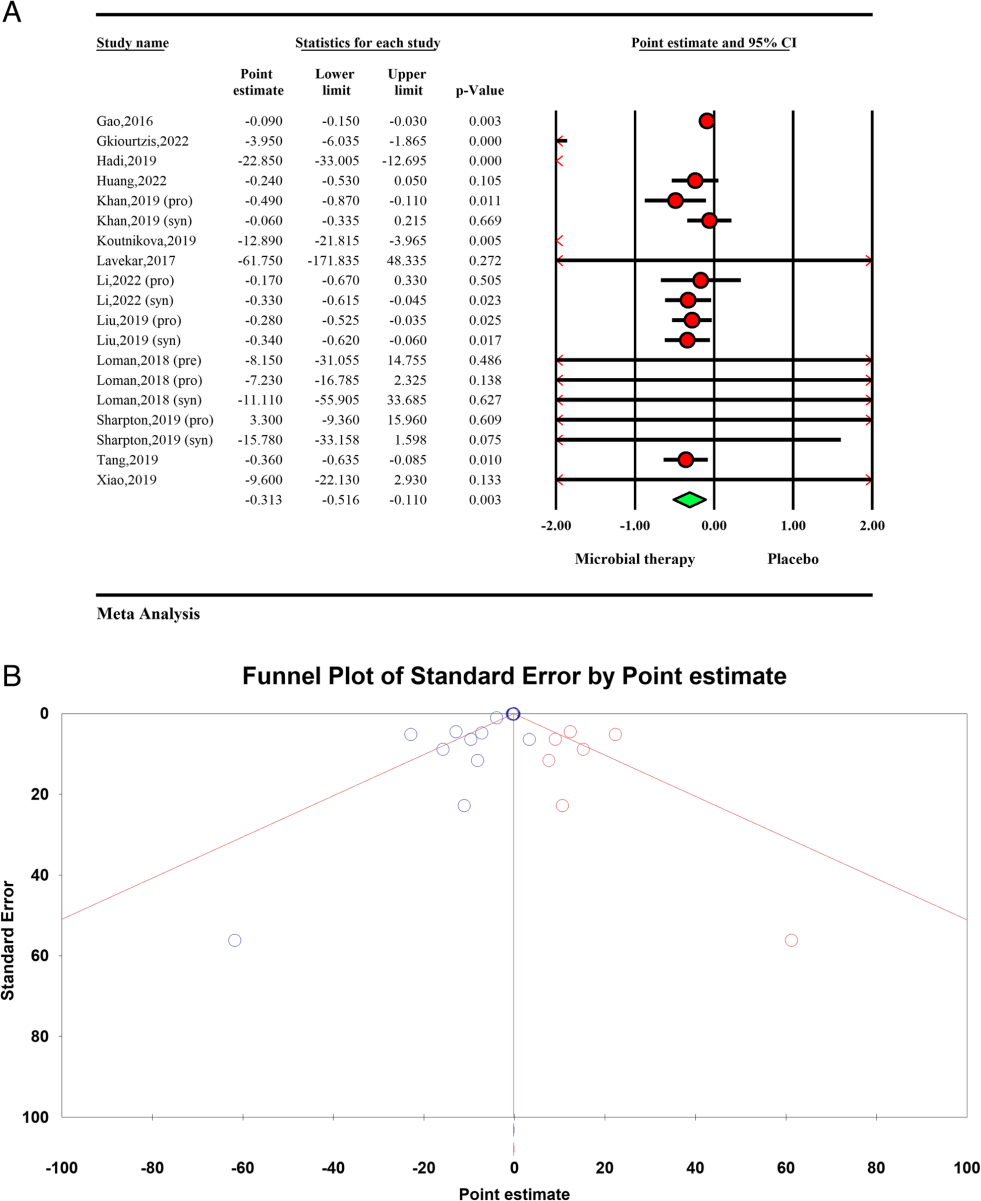
**A** Forest plot for the effect size and 95% confidential interval of microbial therapy on serum TG level in NAFLD patients. **B** Results of publication bias with seven imputed studies (red dots)

The total effect of microbial therapy on serum TG level was significant (ES − 0.31 95%CI − 0.51, − 0.11, *P* < 0.01) with significant heterogeneity within the studies (*I*^2^ = 71.69%, *P* < 0.01) (Fig. 2A). The sensitivity analysis results showed no significant difference in the total effect size after removing each study.

Based on the results of subgroup analysis, studies conducted in China and USA were associated with significant decrease in heterogeneity compared to other countries (*I*^2^ = 44.64%, *P* = 0.08, *I*^2^ = 35.95%, *P* = 0.15, respectively). Moreover, studies with a total sample size above 1000 and that reported their data with standard mean difference (SMD) were associated with decreased heterogeneity. (*I*^2^ = 2.03%, *P* = 0.40, *I*^2^ = 0.00%, *P* = 0.62) (Table 2).

**Table 2:** Results of subgroup analysis with their effect size and 95% confidential interval

**VARIABLE**			**NUMBER OF STUDIES**	**ES WITH 95%CI**	***I***^**2**^	***P* VALUE OF HETEROGENEITY**
**TG**	Total effect		19	(− 0.31: − 0.51, − 0.11, *P* < 0.01)	71.69%	*P* < 0.01
	Intervention type	Probiotics	12	(− 0.32: − 0.56, − 0.08, *P* < 0.01)	69.74%	*P* < 0.01
		Synbiotics	6	(− 0.32: − 0.85, 0.21, *P* = 0.23)	79.88%	*P* < 0.01
		Prebiotics	1	(− 8.15: − 31.05, 14.75, *P* = 0.48)	0.00%	*P* = 1
	Units of reported	MD	2	(− 10.95: − 33.7, 11.17, *P* = 0.33)	94.74%	*P* < 0.01
		SMD	7	(− 0.28: − 0.39, − 0.17, *P* < 0.01)	0.00%	*P* = 0.62
		WMD	10	(− 4.53: − 8.09, − 0.97, *P* = 0.01)	70.70	*P* < 0.01
	Country	China	8	(− 0.13: − 0.18, − 0.08, *P* < 0.01)	44.64%	*P* = 0.08
		USA	7	(− 0.21: − 0.43, 0.00, *P* = 0.05)	35.95%	*P* = 0.15
		Others	4	(− 12.92: − 24.25, − 1.58, *P* = 0.02)	82.19%	*P* < 0.01
	Previous registered protocol	Yes	7	(− 0.58: − 1.15, 0.00, *P* = 0.04)	73.80%	*P* < 0.01
		No	12	(− 0.25: − 0.47, − 0.03, *P* = 0.02)	68.50%	*P* < 0.01
	Quality of studies	Critically low	7	(− 0.21: − 0.37, − 0.05, *P* < 0.01)	50.76%	*P* = 0.05
		Low	7	(− 0.36: − 0.95, 0.23, *P* = 0.23)	72.80%	*P* < 0.01
		High	5	(− 3.60: − 7.42, 0.2, *P* = 0.06)	81.83%	*P* < 0.01
	Fund	Yes	12	(− 0.24: − 0.46, − 0.03, *P* = 0.02)	52.08%	*P* = 0.01
		No	4	(− 0.52: − 1.60, 0.55, *P* = 0.33)	87.24%	*P* < 0.01
		Not reported	3	(− 0.55: − 1.13, 0.02, *P* = 0.06)	82.96	*P* < 0.01
	Sample size	< 500	3	(− 13.98, − 32.17, − 4.19, *P* = 0.13)	85.50%	*P* < 0.01
		500–1000	9	(− 0.23: − 0.42. − 0.03, *P* = 0.01)	58.93%	*P* = 0.01
		< 1000	7	(− 0.30: − 0.45, − 0.14, *P* = < 0.01)	2.03%	*P* = 0.40
**TC**	Total effect		16	(− 1.04: − 1.46, − 0.61, *P* < 0.01)	92.5%	*P* < 0.01
	Intervention type	Probiotics	10	(− 0.37: − 0.58, − 0.15, *P* < 0.01)	72.07%	*P* < 0.01
		Synbiotics	5	(− 5.67; − 8.95, − 2.38, *P* < 0.01)	97.34%	*P* < 0.01
		Prebiotics	1	(− 5.56: − 12.62, 1.5, *P* = 0.12)	0.00%	*P* = 1
	Units of reported	MD	1	(− 17.81: − 25.11, − 10.50, *P* < 0.01)	0.00%	*P* = 1
		SMD	7	(− 0.43: − 0.55, − 0.31, *P* < 0.01)	25.38%	*P* = 0.23
		WMD	8	(− 4.02: − 5.44, − 2.59, *P* < 0.01)	95.71%	*P* < 0.01
	Country	China	9	(− 0.42: − 0.62, − 0.23, *P* < 0.01)	70.58%	*P* < 0.01
		USA	5	(− 5.48: − 10.09, − 0.87, *P* = 0.02)	97.16%	*P* < 0.01
		Others	2	(− 11.57: − 22.97, − 0.17, *P* = 0.04)	86.53%	*P* < 0.01
	Previous registered protocol	Yes	4	(− 0.83; − 1.49, − 0.18, *P* = 0.01)	71.99%	*P* = 0.01
		No	12	(− 1.38: − 2.00, − 0.75, *P* < 0.01)	94.02%	*P* < 0.01
	Quality of studies	Critically low	8	(− 0.35: − 0.57, − 0.12, *P* < 0.01)	66.92%	*P* < 0.01
		Low	6	(− 7.06: − 10.99, − 3.14, *P* < 0.01)	96.88%	*P* < 0.01
		High	2	(− 3.04: − 8.29, 2.21, *P* = 0.25)	84.69%	*P* = 0.01
	Fund	Yes	8	(− 1.80: − 2.58, − 1.02, *P* < 0.01)	95.57%	*P* < 0.01
		No	4	(− 3.69: − 6.70, − 0.67, *P* = 0.01)	90.55%	*P* < 0.01
		Not reported	4	(− 0.44: − 0.77, − 0.10, *P* < 0.01)	65.82%	*P* = 0.03
	Sample size	< 500	4	(− 8.56: − 16.11, − 1.00, *P* = 0.02)	91.78%	*P* < 0.01
		500–1000	8	(− 1.43: − 2.16, − 0.69, *P* < 0.01)	95.27%	*P* < 0.01
		< 1000	4	(− 0.75: − 1.40, − 0.10, *P* = 0.02)	70.91%	*P* < 0.01
**HDL**	Total effect		15	(− 0.06: − 0.19, 0.07, *P* = 0.39)	75.51%	*P* < 0.01
	Intervention type	Probiotics	9	(− 0.14: − 0.28, 0.00, *P* = 0.04)	76.10%	*P* < 0.01
		Synbiotics	5	(0.17:0.00, 0.34, *P* = 0.03)	0.00%	*P* = 0.4
		Prebiotics	1	(2.25:0.68, 3.81, *P* < 0.01)	0.00%	*P* = 1
	Units of reported	MD	2	(− 8.55: − 29.55, 12.43, *P* = 0.42)	93.11%	*P* < 0.01
		SMD	7	(0.00: − 0.19,0.19, *P* = 0.99)	62.83%	*P* = 0.01
		WMD	6	(− 0.14: − 0.32, 0.03, *P* = 0.11)	77.57%	*P* < 0.01
	Country	China	9	(− 0.04: − 0.17, 0.07, *P* = 0.45)	73.22%	*P* < 0.01
		USA	5	(− 0.03: − 0.76, 0.69, *P* = 0.92)	84.67%	*P* < 0.01
		Others	1	(1.54: − 1.42,4.5, *P* = 0.3)	0.00%	*P* = 1
	Previous registered protocol	Yes	3	(0.13: − 0.12,0.39, *P* = 0.29)	60.31%	*P* = 0.08
		No	12	(− 0.14: − 0.30,0.1, *P* = 0.08)	74.56%	*P* < 0.01
	Quality of studies	Critically low	7	(− 0.11: − 0.15, − 0.07, *P* < 0.01)	21.04%	*P* = 0.26
		Low	7	(− 0.01: − 0.69,0.66, *P* = 0.96)	85.44%	*P* < 0.01
		High	1	(0.43: − 0.03,0.89, *P* = 0.06)	0.00%	*P* = 1
	Fund	Yes	9	(− 0.01: − 0.34,0.32, *P* = 0.94)	83.81%	*P* < 0.01
		No	3	(0.03: − 0.43,0.49, *P* = 0.90)	63.75%	*P* = 0.06
		Not reported	3	(− 0.10: − 0.17, − 0.03, *P* < 0.01)	0.00%	*P* = 0.41
	Sample size	< 500	2	(− 0.08: − 0.16, − 0.01, *P* = 0.02)	14.10%	*P* = 0.28
		500–1000	8	(− 0.17: − 0.43,0.08, *P* = 0.17)	74.47%	*P* < 0.01
		< 1000	5	(0.14: − 0.32,0.62, *P* = 0.54)	79.14%	*P* < 0.01
**LDL**	Total effect		15	(− .077: − 1.15, − 0.39, *P* < 0.01)	89.00%	*P* < 0.01
	Intervention type	Probiotics	9	(− 0.22: − 0.27, − 0.18, *P* < 0.01)	47.47%	*P* = 0.05
		Synbiotics	5	(− 3.54: − 5.49, − 1.58, *P* < 0.01)	95.30%	*P* < 0.01
		Prebiotics	1	(− 4.97: − 10.96,1.02, *P* = 0.10)	0.00%	*P* = 1
	Units of reported	MD	2	(− 9.09: − 24.41,6.21, *P* = 0.24)	98.63%	*p* < 0.01
		SMD	7	(− 0.52: − 0.67, − 0.36, *P* < 0.01)	36.95%	*P* = 0.14
		WMD	6	(− 0.21: − 0.26, − 0.16, *P* < 0.01)	32.16%	*P* = 0.19
	Country	China	9	(− 0.53: − 0.76, − 0.31, *P* < 0.01)	72.31%	*P* < 0.01
		USA	5	(− 0.76: − 2.04,0.51, *P* = 0.24)	58.64%	*P* = 0.04
		Others	1	(− 17.01: − 20.50, − 13.52, *P* < 0.01)	0.00%	*P* = 1
	Previous registered protocol	Yes	3	(− 0.53: − 0.77, − 0.30, *P* < 0.01)	0.00%	*P* = 0.37
		No	12	(− 0.96: − 1.49, − 0.42, *P* < 0.01)	90.79%	*P* < 0.01
	Quality of studies	Critically low	7	(− 0.40: − 0.64, − 0.17, *P* < 0.01)	62.84%	*P* = 0.01
		Low	7	(− 3.21: − 4.94, − 1.47, *P* < 0.01)	93.72%	*P* < 0.01
		High	1	(− 0.54: − 0.99; − 0.09. *P* = 0.01)	0.00%	*P* = 1
	Fund	Yes	9	(− 0.59: − 0.94, − 0.24, *P* < 0.01)	66.85%	*P* < 0.01
		No	3	(− 5.28: − 9.42, − 1.15, *P* = 0.01)	97.78%	*P* < 0.01
		Not reported	3	(− 0.51: − 0.71, − 0.31, *P* < 0.01)	41.14%	*P* = 0.18
	Sample size	< 500	2	(− 8.60: − 24.89, 7.69, *P* = 0.30)	98.83%	*P* < 0.01
		500–1000	8	(− 0.44; − 0.75, − 0.13,* P* < 0.01)	66.90%	*P* < 0.01
		< 1000	5	(− 0.61: − 0.83, − 0.38,* P* < 0.01)	39.31%	*P* = 0.15

*MD* Mean difference, *SMD* Standard mean difference, *WMD* Weighted mean difference

Visual inspection of the forest plot showed small study effect, which was confirmed by Egger’s regression test (*p* < 0.001). Further trim and fill analysis with 7 imputes studies showed the result of microbial therapy on TG in NAFLD patients was acceptable (ES =  − 0.30, 95%CI − 0.55, − 0.06) (Fig. 2B).

### Effects of microbial therapy on TC

**Fig. 3:**
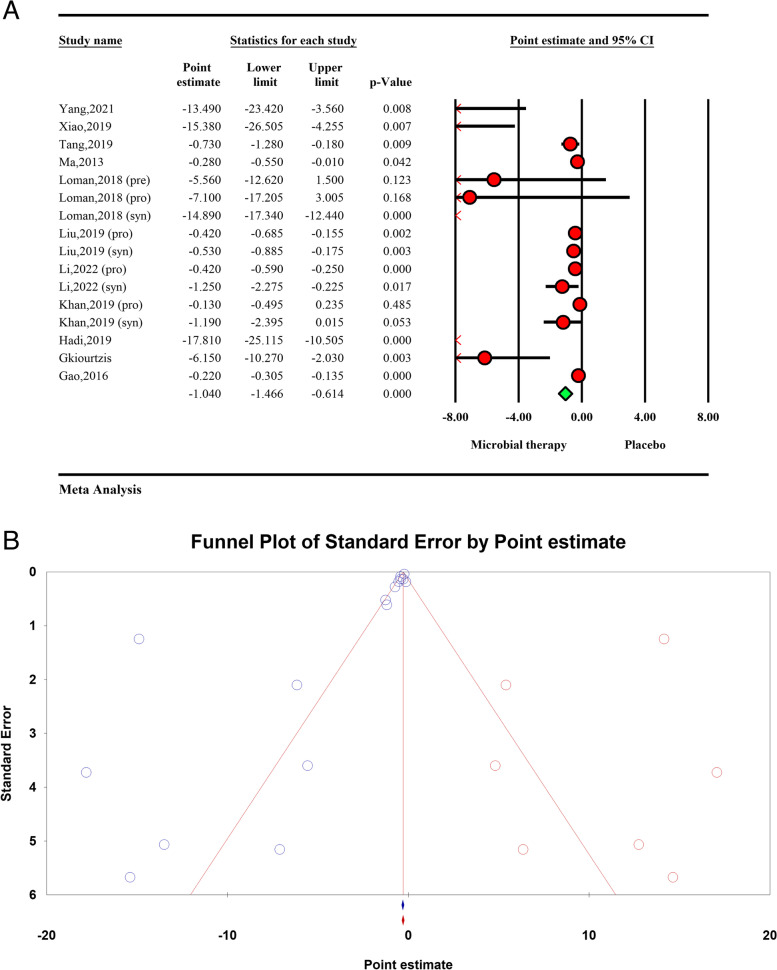
**A** Forest plot for the effect size and 95% confidential interval of microbial therapy on serum TC level in NAFLD patients. **B** Results of publication bias with seven imputed studies (red dots)

The total effect of microbial therapy on decreasing serum TC level was significant (ES − 1.04; − 1.46, − 0.61, *P* < 0.01) (Fig. 3A). Significant heterogeneity was observed among included studies (*I*^2^ = 92.5%, *P* < 0.01).

Results of sensitivity analysis showed elimination of Gao, 2016 and Loman, 2018 (synbiotics) could change the pooled effect (ES − 1.51: − 2.11, − 0.91 *P* < 0.01, ES − 0.50: − 0.75, − 0.25, *P* < 0.01 respectively).

The results of subgroup analysis showed that studies reported their ES in SMD were significantly associated with lower heterogeneity (*I*^2^ = 25.38%, *P* = 0.023) (Table 2). Visual inspection of the funnel plot and Eggers’ regression test showed significant publication bias (*P* < 0.01) and, the ES based on trim and fill analysis with seven imputed studies was -0.50 (95%CI − 1.05, 0.03) (Fig. 3B).

### Effects of microbial therapy on HDL

**Fig. 4:**
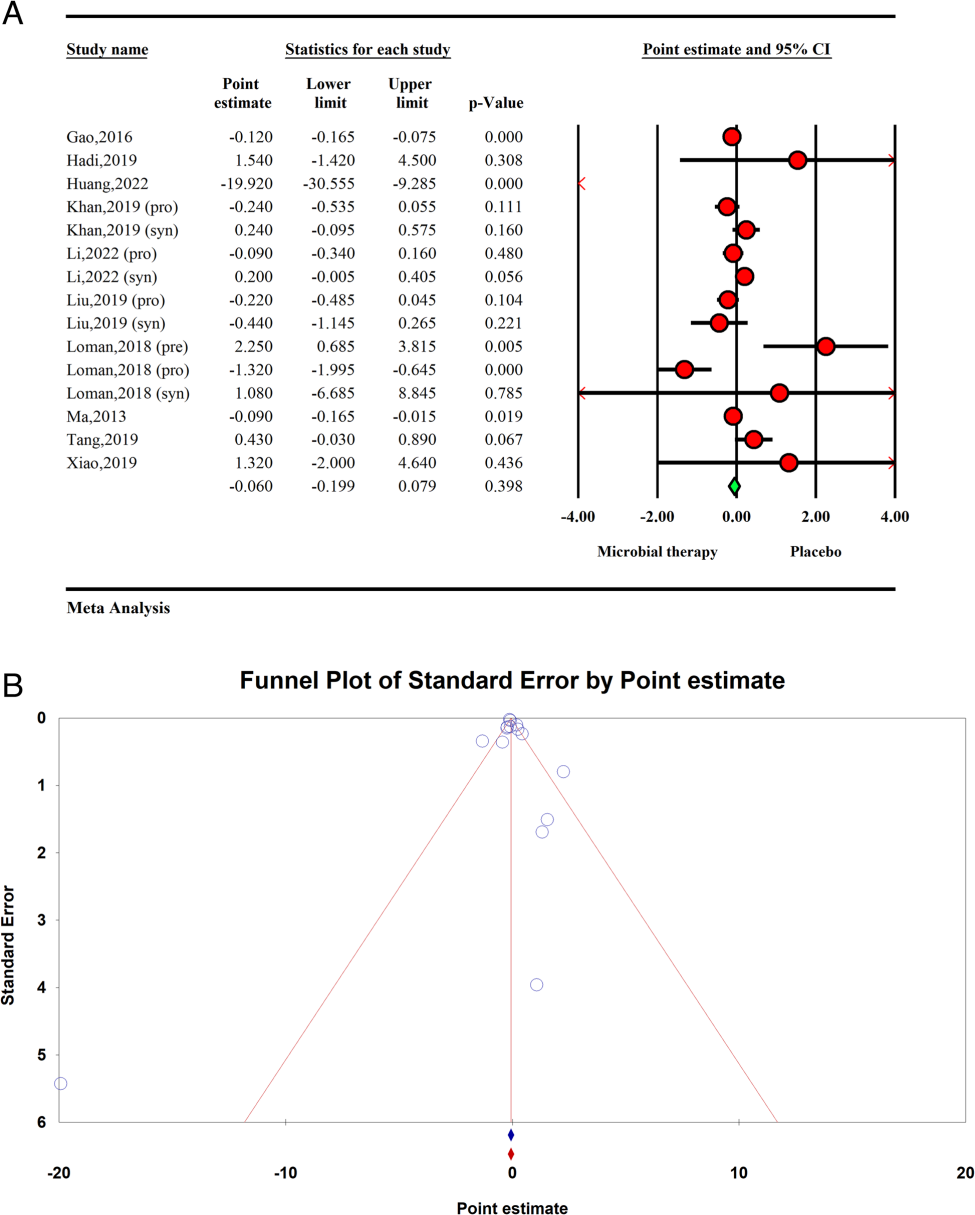
Forest plot for the effect size and 95% confidential interval of microbial therapy on serum HDL level in NAFLD patients (**A**).** B** is illustrating the results of publication bias with two imputed studies (red dots)

The total effect of microbial therapy on serum HDL level was insignificant and heterogenic **(**ES − 0.06; 95% CI − 0.19, 0.07, *P* = 0.39, *I*^2^ = 75.51%, *P* < 0.01) (Fig. 4A). The sensitivity analysis results showed no significant difference in total effect size after the elimination of each study.

The results of subgroup analysis showed studies with synbiotics as intervention, studies with critically low quality, studies with sample sizes less than 500, and studies without reporting their funding source were associated with decreased heterogeneity (*I*^2^ = 0.00%, *P* = 0.4, *I*^2^ = 21.4%, *P* = 0.26, *I*^2^ = 14.10%, *P* = 0.28, *I*^2^ = 0.00%, *P* = 0.41) (Table 2).

Egger’s regression test results showed no publication bias (*P* = 0.77). The ES based on trim and fill analysis with two imputed studies was − 0.05 (95%CI − 0.19, 0.07) (Fig. 4B).

### Effects of microbial therapy on LDL

**Fig. 5:**
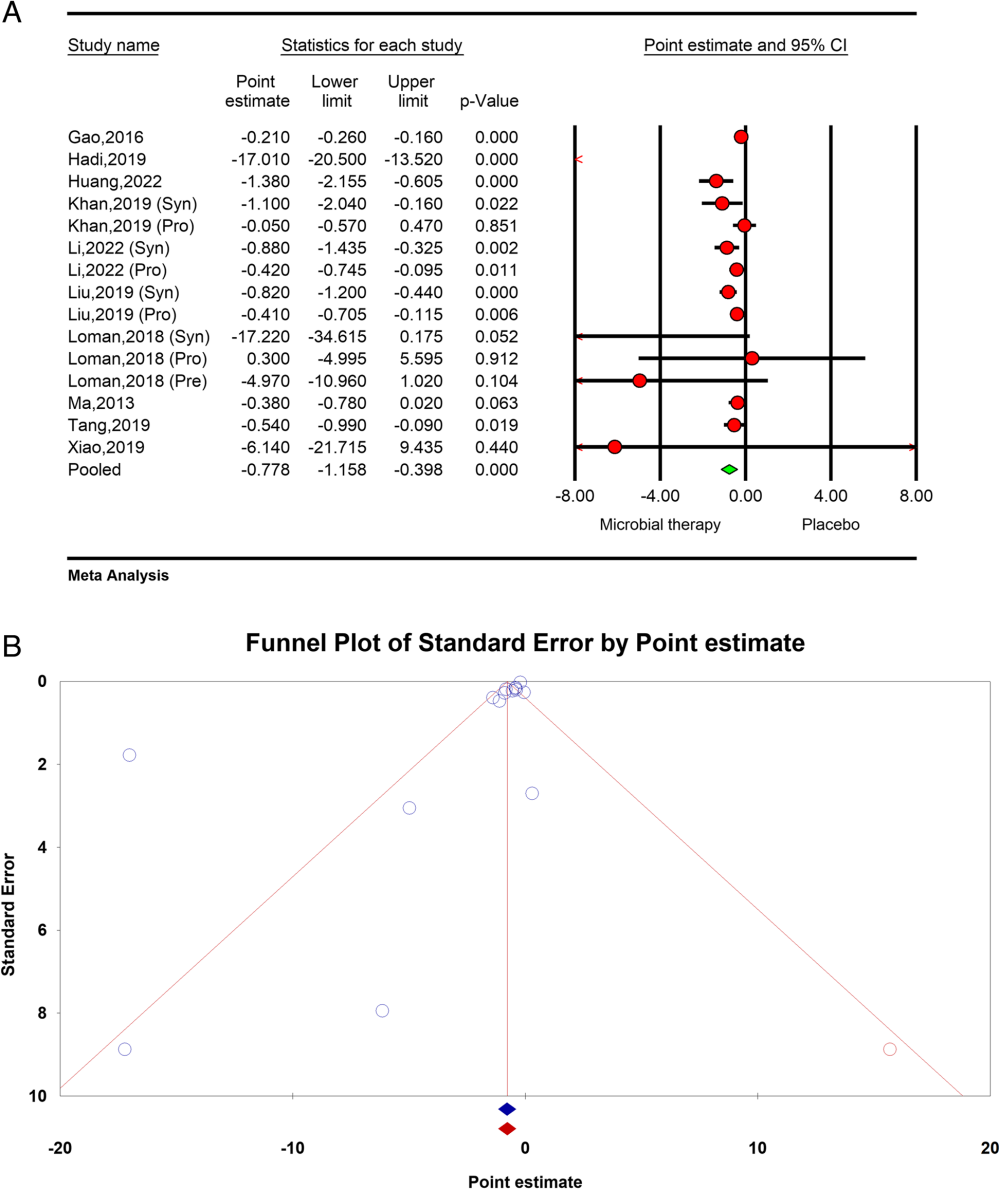
**A** Forest plot for the effect size and 95% confidential interval of microbial therapy on serum LDL level in NAFLD patients.** B** Results of publication bias with one imputed study (red dots)

The total effect of microbial therapy on LDL was significant with great heterogeneity (ES − 0.77; 95%CI − 1.15, − 0.39, *p* < 0.01, *I*^2^ = 89.00%, *P* < 0.01 (Fig. 5A). The results of sensitivity analysis showed that elimination of each study did not affect the pooled effect size.

The results of subgroup analysis showed studies with sample sizes of more than 1000, studies with previously registered protocol, and studies that reported their results in SMD and weighted mean difference (WMD) were accompanied with reduced heterogeneity (*I*^2^ = 39.31%, *P* = 0.15, *I*^2^ = 0.00%, *P* = 0.37, *I*^2^ = 36.95%, *P* = 0.14, *I*^2^ = 32.16%, *P* = 0.19 respectively) (Table 2).

The Eggers’ regression test results showed significant publication bias (*p* < 0.01). The trim and fill analysis results with imputed one study was acceptable (− 0.77; − 1.15, − 0.39) (Fig. 5B).

## Discussion

The effects of microbial therapy on different health-related outcomes have been exclusively studied. Several human and animal studies were conducted the evaluate the impact of gut microbial modulation on liver diseases. Mao et al. reported that the consumption of Costunolide can prevent hepatic damage by regulating gut microbiota [57]. A network pharmacological study by Jiang et al. revealed that Silybum marianum has hepatoprotective effects on patients with NAFLD [58]. This compound is found to be able to regulate gut microbiota [59]. In this umbrella review, we aimed to assess the effects of microbial therapy on lipid profiles in NAFLD individuals. In conclusion, based on 15 meta-analysis studies, we demonstrated that microbial therapy showed promising effects on the lipid profiles of these patients.

In this study, we found that microbial therapy can significantly decrease serum TG levels. However, the results of other meta-analyses were controversial. While in a meta-analysis study by Wang et al., probiotics significantly reduced TG in obese individuals; another meta-analysis by Mo et al., showed that the effect of probiotics on TG in hypercholesterolaemic adults was insignificant [60, 61]. Variability in patient characteristics, different sample sizes and statistical power, and publication bias can cause such controversial results. In the subgroup analysis, we found studies with sample sizes of less than 1000 participants and studies conducted in countries except for the USA and China, and studies reported by mean difference (MD) and WMD were the source of heterogeneity.

In this study, we found that microbial therapy can significantly decrease serum TC levels. This finding was consistent with the results of other studies [62, 63]. Studies that reported their results in units other than SMD were considered the source of heterogeneity.

Based on the results of our study, microbial therapy can significantly decrease serum LDL levels. Other meta-analyses confirm this finding [64, 65]. Regarding serum LDL levels studies that reported their data in MD, studies without previously registered protocol, with sample sizes less than 1000, were considered the source of heterogeneity.

The results of our umbrella review for serum HDL levels revealed that although microbial therapy decreased serum HDL level but it was insignificant. Studies that used probiotics as interventions, with low quality and sample sizes of more than 500, were considered the source of heterogeneity. In subgroup analysis prebiotics and synbiotics could increase serum HDL levels significantly. The results of our study regarding HDL were accompanied with high heterogeneity showing that further RCTs are needed to confirm the conclusion. Other studies reported conflicting results in this regard. Pan et al. showed the effect of probiotics on Serum HDL levels was not statistically significant [66]. Another meta-analysis by Cho et al. on 30 RCTs with 1624 individuals showed no significant effects of probiotics on serum HDL levels [64]. Mo et al. in a meta-analysis study revealed no significant effects of probiotics in hypercholesteremic patients [61]. Kocsis et al. and Hu et al. in their meta-analysis studies reported significant effects of probiotics on HDL in patients with type 2 diabetes mellitus [67, 68].

One crucial aspect that requires consideration is our database search. While we thoroughly searched multiple databases, it is important to note that we did not include EMBASE in our search. Surprisingly, this database was included in 12 other studies that we analyzed. Consequently, one possible reason for the variations between our study’s results and those of previously published research lies in the differences in the databases searched.

The mechanisms of how microbial therapy can enhance lipid profiles in NALFD patients are complicated and need to be fully understood. Some postulated mechanisms are: bile salt deconjugation, increased LDL hepatic receptors, increased bile salt excretion, co-precipitation of cholesterol, assimilation of cholesterol and bile salt into the probiotics cell membrane, cholesterol reduction, inhibition of Niemann–Pick C1 like 1 expression, and hepatic synthesis of cholesterol inhibition. The mentioned mechanisms will be discussed more details.

### 1-Bile salt deconjugation and 2-increased hepatic LDL receptors

**Fig. 6:**
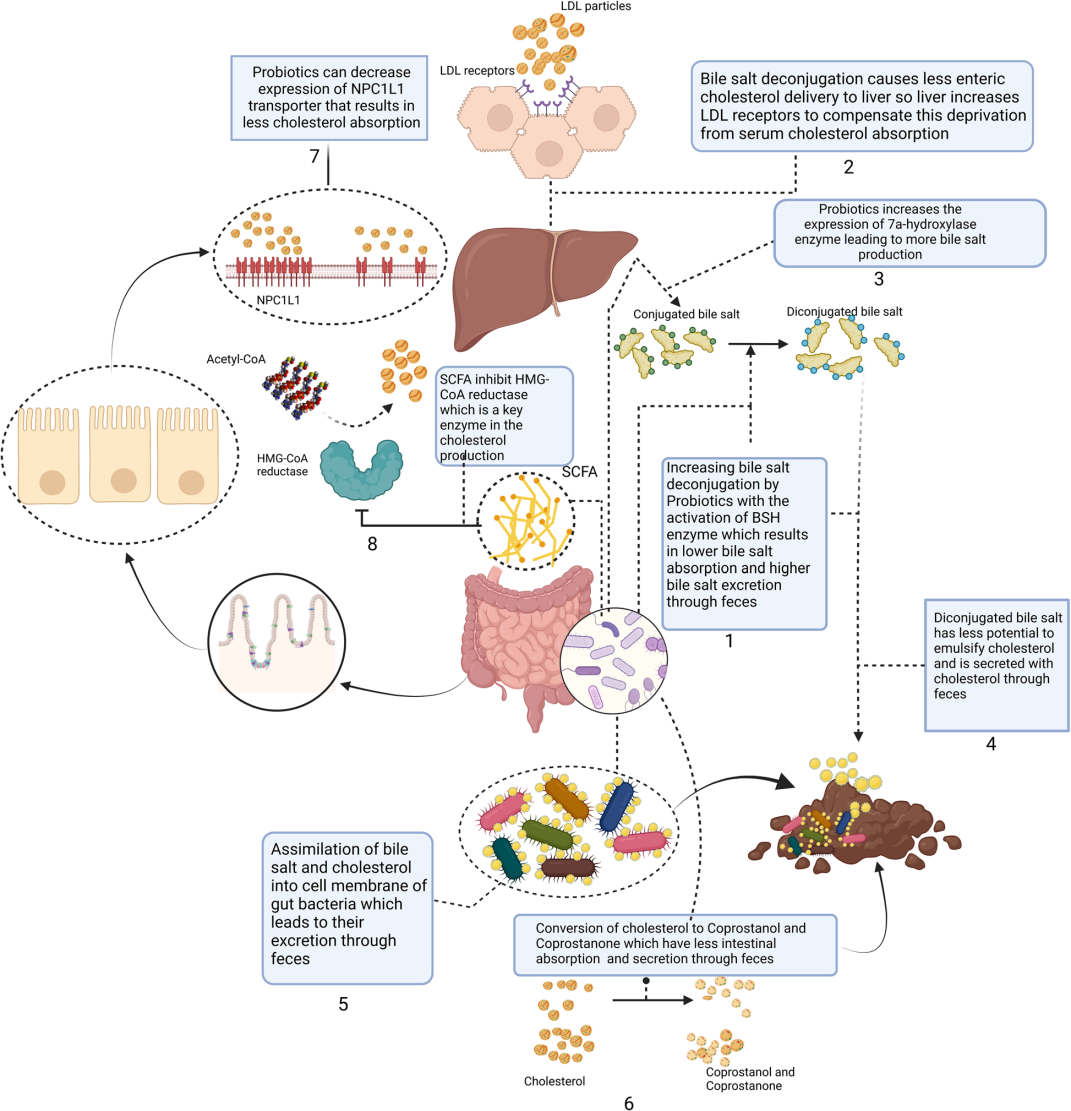
Mechanism of gut microbiome modulation on lipid profile: 1 Bile salt deconjugation. 2 Increased hepatic LDL receptors. 3 Increased bile salt excretion. 4 Co-precipitation of cholesterol. 5 Assimilation of cholesterol and bile salt into the probiotics cell membrane. 6 Cholesterol reduction. 7 Inhibition of Niemann–Pick C1 like 1 expression. 8 Hepatic synthesis of cholesterol inhibition

Bile salt plays an essential role in the digestion process. A significant part of bile salt in the intestinal lumen is reabsorbed through the enterohepatic cycle, but 400 to 800 mg of bile salt will remain in the intestinal lumen, which can be deconjugated by gut microbiota [69, 70]. This deconjugation process is done by the activity of an enzyme called bile salt hydrolase (BSH) [70]. Deconjugated bile is more efficient for gut microbiota replication as conjugated bile salt has anti-bacterial properties [71]. Deconjugated bile salt has lower solubility, resulting in lower bile reabsorption and higher bile salt excretion with feces [71]. Lower absorption of bile salt from intestinal barriers results in lower cholesterol delivery to the liver, which is needed for denovo synthesis; hence liver compensates for this deprivation by increasing hepatocyte LDL receptor and absorption of serum LDL, which results in lower serum LDL concentration [72]. Probiotics are considered to have positive BSH effects; hence their administration can lower serum TC levels [73] (Fig. 6).

### 3-Increased bile salt excretion

It is hypothesized that probiotics can increase the expression of 7a-hydroxylase (CYP7A1), an enzyme in bile salt synthesis. As discussed, this bile salt can be converted into deconjugated form [74]. Increased bile salt synthesis, along with its deconjugation, results in higher bile salt with the containing cholesterol excretion through feces (Fig. 6).

### 4-Co-precipitation of cholesterol

The absorption of diet cholesterol by enterocytes occurs via the hydrophobic surface of cholesterol; thus, cholesterol needs an emulsifier for its absorption, and bile salt is the emulsifier of cholesterol [70]. As previously discussed, bile salt is deconjugated by the effects of probiotics. Deconjugated bile salt has less potential to act as an emulsifier for cholesterol absorption; hence absorption of lipid particles decreases [75, 76] (Fig. 6).

### 5-Assimilation of cholesterol and bile salt into the probiotics cell membrane

The cholesterol content of the medium can be assimilated into the cell membrane of probiotics and be secreted via feces [77]. As a consequence of this process, bacterial membrane composition is changed, leading to higher resistance of probiotics in the intestinal environment [71, 78]. The assimilation of cholesterol into probiotics cell membrane can be facilitated by deconjugated bile salt [79] (Fig. 6).

### 6-Cholesterol reduction

The cholesterol content of the medium can be transformed into coprostanol, and in lesser amounts, to coprostanone. This transformation is dependent on an enzyme activity called cholesterol reductase. Some probiotics have cholesterol reducing properties. Coprostanol and coprostanone have less intestinal absorption and are eliminated via feces [70] (Fig. 6).

### 7-Inhibition of Niemann–Pick C1 like 1 expression

Cholesterol particles of the medium are absorbed via Niemann–Pick C1 like 1 (NPC1L1) that are transporters located on intestinal cells membrane [80, 81]. Previous in vitro studies showed some probiotics could reduce NPC1L1 expression on the cellular surface and consequently decrease cholesterol absorption [82, 83] (Fig. 6).

### 8-Hepatic synthesis of cholesterol inhibition

Short-chain fatty acids are the products of probiotics from the fermentation of non-digestible carbohydrates [84]. Some sorts of SCFA, like propionate, have the potential to inhibit the enzyme 3-hydroxy-3-methylglutaryl coenzyme A (HMG-CoA) reductase, which plays an essential role in the hepatic cholesterol synthesis process [85] (Fig. 6).

## Advantages, limitations, and future research

This umbrella meta-analysis study showed how gut microbial therapy could modify lipid profiles in NAFLD individuals. The results were promising for LDL, TC, and TG; however, microbial therapy did not have significant effects regarding HDL. Our results shed light on the treatment of NAFLD as microbial therapy is cheap, safe, and without toxin substrate accumulation compared to other therapeutic drugs [86, 87].

Our study had some limitations; first, we could not determine the optimum dosage and duration of treatment of microbial therapy since the number of meta-analyses discussed dosage and duration was insufficient. Second, we did not assess how microbial therapy should be administered, whether in capsule drugs or additional supplements to the diet. Some of the mechanisms we proposed for how microbial therapy can modulate lipid profile were observed in animal studies, and more human clinical trials are needed to prove them. We highly recommend future meta-analyses to conduct sub group analysis based on the quality of included studies and funding sources, as most of the meta-analyses in this umbrella review did not perform such subgrouping. We suggest that researchers perform clinical trials with prebiotics and synbiotics, as most studies administered probiotics.

## Conclusion

In conclusion, this umbrella review on the meta-analyses of randomized control trials provided insights into the impact of gut microbial therapy, including probiotics, synbiotics, and prebiotics, on the lipid profile of individuals with NAFLD. The findings of this umbrella review suggested that microbial therapy has positive effects on the lipid profile parameters in individuals with NAFLD. The analysis revealed a significant reduction in TG, TC, and LDL levels following microbial therapy intervention. These results indicated the potential of microbial therapy as an effective intervention for improving the lipid profile in NAFLD patients. Overall, these findings contribute to the growing body of evidence supporting the use of microbial therapy as a promising approach for managing metabolic disorders and improving lipid profiles. However, more well-designed randomized control trials are needed to further validate these results and determine the optimal regimen, duration, and specific microbial strains that yield the most significant benefits.

### Supplementary Information

**Additional file 1: Table S1.** Search strategy and keywords of this umbrella review. **Table S2.** Quality assessment of included studies based on AMSTAR 2 checklist

## Data Availability

The datasets used and/or analyzed during the current study are available from the corresponding author on reasonable request.

## Abbreviations

PRISMA: *Preferred Reporting Items for Systematic Reviews and Meta-Analyses*
RCT: Randomized control trial
TG: Triglyceride
HDL: High-density lipoprotein
LDL: Low-density lipoprotein
TC: Total cholesterol
NAFLD: Non-alcoholic fatty liver disease
ES: Effect size
MD: Mean difference
WMD: Weighted mean difference
SMD: Standard mean difference

## References

[1] Puri P, Sanyal AJ (2012). Nonalcoholic fatty liver disease: Definitions, risk factors, and workup. Clinical liver disease.

[2] Abd El-Kader SM, El-Den Ashmawy EM (2015). Non-alcoholic fatty liver disease: the diagnosis and management. World J Hepatol.

[3] Angulo P, Machado MV, Diehl AM (2015). Fibrosis in nonalcoholic fatty liver disease: mechanisms and clinical implications. Semin Liver Dis.

[4] Cooper J, Baumgartner K, Smith A, St LJ (2021). Liver disease: nonalcoholic fatty liver disease. FP essentials.

[5] Tevar AD, Clarke C, Wang J (2010). Clinical review of nonalcoholic steatohepatitis in liver surgery and transplantation. J Am Coll Surg.

[6] Collier J (2007). Non-alcoholic fatty liver disease. Medicine.

[7] Paschos P, Paletas K (2009). Non alcoholic fatty liver disease and metabolic syndrome. Hippokratia.

[8] Moore JB (2010). Non-alcoholic fatty liver disease: the hepatic consequence of obesity and the metabolic syndrome. Proceedings of the Nutrition Society.

[9] Le MH, Yeo YH, Li X, et al. 2019 Global NAFLD Prevalence: A Systematic Review and Meta-analysis. Clin Gastroenterol Hepatol. 2021. 10.1016/j.cgh.2021.12.002. 10.1016/j.cgh.2021.12.002 34890795

[10] Cheemerla S, Balakrishnan M (2021). Global epidemiology of chronic liver disease. Clin Liver Dis.

[11] Koopman N, Molinaro A, Nieuwdorp M, Holleboom AG (2019). can bugs be drugs? The potential of probiotics and prebiotics as treatment for non-alcoholic fatty liver disease. Aliment Pharmacol Ther.

[12] Sookoian S, Pirola CJ (2017). Genetic predisposition in nonalcoholic fatty liver disease. Clin Mol Hepatol.

[13] Mokhtari Z, Gibson DL, Hekmatdoost A (2017). Nonalcoholic fatty liver disease, the gut microbiome, and diet. Adv Nutr.

[14] Meroni M, Longo M, Dongiovanni P (2019). The role of probiotics in nonalcoholic fatty liver disease: a new insight into therapeutic strategies. Nutrients.

[15] Moschen AR, Kaser S, Tilg H (2013). Non-alcoholic steatohepatitis: a microbiota-driven disease. Trends Endocrinol Metab.

[16] Tripathi A, Debelius J, Brenner DA (2018). The gut–liver axis and the intersection with the microbiome. Nat Rev Gastroenterol Hepatol.

[17] Cao Y, Deng Y, Wang J, Zhao H, Zhang J, Xie W (2021). The association between NAFLD and risk of chronic kidney disease: a cross-sectional study. Therapeutic Adv Chronic Dis.

[18] Ma C, Yan K, Wang Z (2021). The association between hypertension and nonalcoholic fatty liver disease (NAFLD): literature evidence and systems biology analysis. Bioengineered.

[19] Targher G, Corey KE, Byrne CD, Roden M (2021). The complex link between NAFLD and type 2 diabetes mellitus — mechanisms and treatments. Nat Rev Gastroenterol Hepatol..

[20] Fabbrini E, Sullivan S, Klein S (2010). Obesity and nonalcoholic fatty liver disease: biochemical, metabolic, and clinical implications. Hepatology (Baltimore, MD).

[21] Arslan U, Yenerçağ M (2020). Relationship between non-alcoholic fatty liver disease and coronary heart disease. World J Clin Cases.

[22] Paschos P, Paletas K (2009). Non alcoholic fatty liver disease and metabolic syndrome. Hippokratia.

[23] Amini-Salehi E, Hassanipour S, Joukar F (2023). Risk factors of non-alcoholic fatty liver disease in the iranian adult population: a systematic review and meta-analysis. Systematic Review. Hepat Mon..

[24] Phisalprapa P, Prasitwarachot R, Kositamongkol C (2021). Economic burden of non-alcoholic steatohepatitis with significant fibrosis in Thailand. BMC Gastroenterol..

[25] Allen AM, Van Houten HK, Sangaralingham LR, Talwalkar JA, McCoy RG (2018). Healthcare cost and utilization in nonalcoholic fatty liver disease: real-world data from a large U.S. Claims Database. Hepatology (Baltimore, Md).

[26] Younossi Z, Anstee QM, Marietti M (2018). Global burden of NAFLD and NASH: trends, predictions, risk factors and prevention. Nat Rev Gastroenterol Hepatol.

[27] Pouwels S, Sakran N, Graham Y (2022). Non-alcoholic fatty liver disease (NAFLD): a review of pathophysiology, clinical management and effects of weight loss. BMC Endocr Disord.

[28] Zhou Y, Zheng T, Chen H (2018). Microbial intervention as a novel target in treatment of non-alcoholic fatty liver disease progression. Cell Physiol Biochem.

[29] Meroni M, Longo M, Dongiovanni P. The role of probiotics in nonalcoholic fatty liver disease: a new insight into therapeutic strategies. Nutrients. 2019;11(11). 10.3390/nu11112642. 10.3390/nu11112642 PMC6893730 31689910

[30] Lynch SV, Pedersen O (2016). The human intestinal microbiome in health and disease. N Engl J Med.

[31] Davani-Davari D, Negahdaripour M, Karimzadeh I, et al. Prebiotics: definition, types, sources, mechanisms, and clinical applications. Foods (Basel, Switzerland). 2019;8(3). 10.3390/foods8030092. 10.3390/foods8030092 PMC6463098 30857316

[32] de Vrese M, Schrezenmeir J (2008). Probiotics, prebiotics, and synbiotics. Adv Biochem Eng Biotechnol.

[33] Koutnikova H, Genser B, Monteiro-Sepulveda M (2019). Impact of bacterial probiotics on obesity, diabetes and non-alcoholic fatty liver disease related variables: a systematic review and meta-analysis of randomised controlled trials. BMJ Open.

[34] Xiao M-W, Lin S-X, Shen Z-H, Luo W-W, Wang X-Y. Systematic review with meta-analysis: the effects of probiotics in nonalcoholic fatty liver disease. Gastroenterol Res Pract. 2019;2019:14845982019. 10.1155/2019/1484598 PMC6927028 31885541

[35] Khalesi S, Johnson DW, Campbell K (2018). Effect of probiotics and synbiotics consumption on serum concentrations of liver function test enzymes: a systematic review and meta-analysis. Eur J Nutr.

[36] Hadi A, Mohammadi H, Miraghajani M, Ghaedi E (2019). Efficacy of synbiotic supplementation in patients with nonalcoholic fatty liver disease: a systematic review and meta-analysis of clinical trials: Synbiotic supplementation and NAFLD. Crit Rev Food Sci Nutr.

[37] Stachowska E, Portincasa P, Jamioł-Milc D, Maciejewska-Markiewicz D, Skonieczna-Żydecka K. The relationship between prebiotic supplementation and anthropometric and biochemical parameters in patients with NAFLD-A systematic review and meta-analysis of randomized controlled trials. Nutrients. 2020;12(11). 10.3390/nu12113460. 10.3390/nu12113460 PMC7698299 33187278

[38] Shuster J. Review: Cochrane handbook for systematic reviews for interventions, Version 5.1.0, published 3/2011. Julian P.T. Higgins and Sally Green, Editors. Res Synth Methods. 2011;2:126–30.

[39] Page MJ, McKenzie JE, Bossuyt PM (2021). The PRISMA 2020 statement: an updated guideline for reporting systematic reviews. Syst Rev.

[40] Shea BJRB, Wells G, Thuku M, Hamel C, Moran J, Moher D, Tugwell P, Welch V, Kristjansson E, Henry DA (2017). AMSTAR 2: a critical appraisal tool for systematic reviews that include randomised or non-randomised studies of healthcare interventions, or both. BMJ..

[41] Sterne JA, Sutton AJ, Ioannidis JP (2011). Recommendations for examining and interpreting funnel plot asymmetry in meta-analyses of randomised controlled trials. Bmj..

[42] Egger M, Davey Smith G, Schneider M, Minder C (1997). Bias in meta-analysis detected by a simple, graphical test. BMJ.

[43] Gkiourtzis Ν, Kalopitas G, Vadarlis A (2022). The benefit of probiotics in pediatric nonalcoholic fatty liver disease: a meta-analysis of randomized control trials. J Pediatr Gastroenterol Nutr.

[44] Huang Y, Wang X, Zhang L (2022). Effect of probiotics therapy on nonalcoholic fatty liver disease. Comput Math Methods Med.

[45] Li S, Liu J, Wang Z (2022). The promising role of probiotics/prebiotics/synbiotics in energy metabolism biomarkers in patients with NAFLD: a systematic review and meta-analysis. Front Public Health..

[46] Yang R, Shang J, Zhou Y, Liu W, Tian Y, Shang H (2021). Effects of probiotics on nonalcoholic fatty liver disease: a systematic review and meta-analysis. Expert Rev Gastroenterol Hepatol.

[47] Koutnikova H, Genser B, Monteiro-Sepulveda M (2019). Impact of bacterial probiotics on obesity, diabetes and non-alcoholic fatty liver disease related variables: a systematic review and meta-analysis of randomised controlled trials. BMJ Open..

[48] Xiao MW, Lin SX, Shen ZH, Luo WW, Wang XY (2019). Systematic review with meta-analysis: the effects of probiotics in nonalcoholic fatty liver disease. Gastroenterol Res Pract.

[49] Liu L, Li P, Liu Y, Zhang Y (2019). Efficacy of probiotics and synbiotics in patients with nonalcoholic fatty liver disease: a meta-analysis. Dig Dis Sci.

[50] Khan MY, Mihali AB, Rawala MS, Aslam A, Siddiqui WJ (2019). The promising role of probiotic and synbiotic therapy in aminotransferase levels and inflammatory markers in patients with nonalcoholic fatty liver disease - a systematic review and meta-analysis. Eur J Gastroenterol Hepatol.

[51] Sharpton SR, Maraj B, Harding-Theobald E, Vittinghoff E, Terrault NA (2019). Gut microbiome-targeted therapies in nonalcoholic fatty liver disease: a systematic review, meta-analysis, and meta-regression. Am J Clin Nutr.

[52] Tang Y, Huang J, Zhang WY (2019). Effects of probiotics on nonalcoholic fatty liver disease: a systematic review and meta-analysis. Therap Adv Gastroenterol.

[53] Loman BR, Hernández-Saavedra D, An R, Rector RS (2018). Prebiotic and probiotic treatment of nonalcoholic fatty liver disease: a systematic review and meta-analysis. Nutr Rev.

[54] A SL, D VR, Manohar T, A AL. Role of probiotics in the treatment of nonalcoholic fatty liver disease: a meta-analysis. Euroasian J Hepatogastroenterol. 2017;7(2):130–137. 10.5005/jp-journals-10018-1233. 10.5005/jp-journals-10018-1233 PMC5670255 29201794

[55] Gao X, Zhu Y, Wen Y, Liu G, Wan C (2016). Efficacy of probiotics in non-alcoholic fatty liver disease in adult and children: a meta-analysis of randomized controlled trials. Hepatol Res.

[56] Ma YY, Li L, Yu CH, Shen Z, Chen LH, Li YM (2013). Effects of probiotics on nonalcoholic fatty liver disease: a meta-analysis. World J Gastroenterol.

[57] Mao J, Zhan H, Meng F (2022). Costunolide protects against alcohol-induced liver injury by regulating gut microbiota, oxidative stress and attenuating inflammation in vivo and in vitro. Phytother Res.

[58] Jiang G, Sun C, Wang X (2022). Hepatoprotective mechanism of Silybum marianum on nonalcoholic fatty liver disease based on network pharmacology and experimental verification. Bioengineered.

[59] Xu S, Jiang X, Jia X, et al. Silymarin modulates microbiota in the gut to improve the health of sow from late gestation to lactation. Animals (Basel). 2022;12(17). 10.3390/ani12172202. 10.3390/ani12172202 PMC9454421 36077922

[60] Wang C, Li S, Xue P (2021). The effect of probiotic supplementation on lipid profiles in adults with overweight or obesity: A meta-analysis of randomized controlled trials. Journal of Functional Foods..

[61] Mo R, Zhang X, Yang Y (2019). Effect of probiotics on lipid profiles in hypercholesterolaemic adults: a meta-analysis of randomized controlled trials. Med Clin (Barc).

[62] Jiang J, Wu C, Zhang C (2020). Effects of probiotic supplementation on cardiovascular risk factors in hypercholesterolemia: A systematic review and meta-analysis of randomized clinical trial. J Functional Foods..

[63] Behrouz V, Aryaeian N, Zahedi MJ, Jazayeri S (2020). Effects of probiotic and prebiotic supplementation on metabolic parameters, liver aminotransferases, and systemic inflammation in nonalcoholic fatty liver disease: A randomized clinical trial. J Food Sci.

[64] Cho YA, Kim J (2015). Effect of probiotics on blood lipid concentrations: a meta-analysis of randomized controlled trials. Medicine (Baltimore)..

[65] Wu Y, Zhang Q, Ren Y, Ruan Z (2017). Effect of probiotic Lactobacillus on lipid profile: a systematic review and meta-analysis of randomized, controlled trials. PLoS One..

[66] Pan B, Liu X, Shi J (2021). A meta-analysis of microbial therapy against metabolic syndrome: evidence from randomized controlled trials. Front Nutr..

[67] Kocsis T, Molnár B, Németh D (2020). Probiotics have beneficial metabolic effects in patients with type 2 diabetes mellitus: a meta-analysis of randomized clinical trials. Sci Rep..

[68] Hu YM, Zhou F, Yuan Y, Xu YC (2017). Effects of probiotics supplement in patients with type 2 diabetes mellitus: a meta-analysis of randomized trials. Med Clin (Barc)..

[69] Ridlon JM, Kang DJ, Hylemon PB (2006). Bile salt biotransformations by human intestinal bacteria. J Lipid Res.

[70] Gérard P (2013). Metabolism of cholesterol and bile acids by the gut microbiota. Pathogens.

[71] Kumar M, Nagpal R, Kumar R (2012). Cholesterol-lowering probiotics as potential biotherapeutics for metabolic diseases. Exp Diabetes Res..

[72] Lecerf JM, de Lorgeril M (2011). Dietary cholesterol: from physiology to cardiovascular risk. Br J Nutr.

[73] Ishimwe N, Daliri EB, Lee BH, Fang F, Du G (2015). The perspective on cholesterol-lowering mechanisms of probiotics. Mol Nutr Food Res.

[74] Zhao C, Dahlman-Wright K (2010). Liver X receptor in cholesterol metabolism. J Endocrinol.

[75] Fava F, Lovegrove JA, Gitau R, Jackson KG, Tuohy KM (2006). The gut microbiota and lipid metabolism: implications for human health and coronary heart disease. Curr Med Chem.

[76] Ahn YT, Kim GB, Lim KS, Baek YJ, Kim HU (2003). Deconjugation of bile salts by Lactobacillus acidophilus isolates. Int Dairy J..

[77] Lye HS, Kuan CY, Ewe JA, Fung WY, Liong MT (2009). The improvement of hypertension by probiotics: effects on cholesterol, diabetes, renin, and phytoestrogens. Int J Mol Sci.

[78] Lye HS, Rusul G, Liong MT (2010). Removal of cholesterol by lactobacilli via incorporation and conversion to coprostanol. J Dairy Sci.

[79] Lye H-S, Rahmat-Ali G, Liong M-T (2010). Mechanisms of cholesterol removal by lactobacilli under conditions that mimic the human gastrointestinal tract. Int Dairy J..

[80] Davis HR, Zhu LJ, Hoos LM (2004). Niemann-Pick C1 Like 1 (NPC1L1) is the intestinal phytosterol and cholesterol transporter and a key modulator of whole-body cholesterol homeostasis. J Biol Chem.

[81] Altmann SW, Davis HR, Zhu LJ (2004). Niemann-Pick C1 Like 1 protein is critical for intestinal cholesterol absorption. Science.

[82] Huang Y, Wu F, Wang X, Sui Y, Yang L, Wang J (2013). Characterization of Lactobacillus plantarum Lp27 isolated from Tibetan kefir grains: a potential probiotic bacterium with cholesterol-lowering effects. J Dairy Sci.

[83] Huang Y, Zheng Y (2010). The probiotic Lactobacillus acidophilus reduces cholesterol absorption through the down-regulation of Niemann-Pick C1-like 1 in Caco-2 cells. Br J Nutr.

[84] Guilloteau P, Martin L, Eeckhaut V, Ducatelle R, Zabielski R, Van Immerseel F (2010). From the gut to the peripheral tissues: the multiple effects of butyrate. Nutr Res Rev.

[85] Wolever TM, Spadafora PJ, Cunnane SC, Pencharz PB (1995). Propionate inhibits incorporation of colonic [1,2–13C]acetate into plasma lipids in humans. Am J Clin Nutr.

[86] Bordoni A, Amaretti A, Leonardi A (2013). Cholesterol-lowering probiotics: in vitro selection and in vivo testing of bifidobacteria. Appl Microbiol Biotechnol.

[87] Stancu CS, Sanda GM, Deleanu M, Sima AV (2014). Probiotics determine hypolipidemic and antioxidant effects in hyperlipidemic hamsters. Mol Nutr Food Res.

