# Supplementary material for: Effects of gut microbial therapy on lipid profile in individuals with non-alcoholic fatty liver disease: an umbrella meta-analysis study
Source: Syst Rev. 2023 Aug 21;12:144. doi: 10.1186/s13643-023-02299-x (PMC10441764; doi:10.1186/s13643-023-02299-x)
Supplement: Supplementary file 1 — Additional file 1: Table S1. Search strategy and keywords of this umbrella review. Table S2. Quality assessment of included studies based on AMSTAR 2 checklist [file 13643_2023_2299_MOESM1_ESM.docx]

**Table S1: Search strategy and keywords of this umbrella review**

| Search query | Keywords (searched through titles, abstracts, medical subject heading (MeSH), and general keywords) |
| --- | --- |
| Query 1 | ( "Non alcoholic Fatty Liver Disease"  OR  "NAFLD"  OR  " Nonalcoholic Fatty Liver Disease"  OR  "Fatty Liver, Nonalcoholic"  OR  "Fatty Livers, Nonalcoholic"  OR  "Liver, Nonalcoholic Fatty"  OR  "Livers, Nonalcoholic Fatty"  OR  "Nonalcoholic Fatty Liver"  OR  "Nonalcoholic Fatty Livers"  OR  "Nonalcoholic Steatohepatitis"  OR  "Nonalcoholic Steatohepatitides"  OR  "Steatohepatitides, Nonalcoholic"  OR  "Steatohepatitis, Nonalcoholic" ) |
| Query 2 | ( "Prebiotics"  OR  "Probiotics"  OR  "Synbiotics" OR "Enterococcus" or "Lactobacillus" or "Bacillus bifidus" ) |
| Query 3 | ( "systematic reviews"  OR  "meta-analysis" ) |
| Final  search  query | Queries 1 AND 2 AND 3 |

**Table S2: Quality assessment of included studies based on AMSTAR 2 checklist**

| **Study name** | **Q1** | **Q2** | **Q3** | **Q4** | **Q5** | **Q6** | **Q7** | **Q8** | **Q9** | **Q10** | **Q11** | **Q12** | **Q13** | **Q14** | **Q15** | **Q16** | **Total quality** |
| --- | --- | --- | --- | --- | --- | --- | --- | --- | --- | --- | --- | --- | --- | --- | --- | --- | --- |
| **Tang,2019** | YES | YES | Yes | Partial yes | YES | YES | Partial Yes | YES | YES | No | YES | Yes | Yes | Yes | Yes | YES | High quality |
| **Gao,2016** | YES | No | Yes | Yes | YES | YES | Partial Yes | YES | YES | No | YES | Yes | Yes | Yes | No | YES | Critically Low quality |
| **MA,2013** | YES | No | Yes | Partial yes | YES | YES | No | No | YES | No | YES | Yes | Yes | No | No | YES | Critically Low quality |
| **Hadi,2019** | YES | No | Yes | Yes | YES | YES | Partial Yes | YES | YES | No | YES | Yes | Yes | Yes | Yes | YES | Low quality |
| **Sharpton,2019** | YES | YES | Yes | Yes | YES | YES | Yes | YES | YES | No | YES | Yes | Yes | Yes | Yes | YES | High quality |
| **Khan,2019** | YES | No | Yes | Partial yes | YES | YES | Partial Yes | YES | YES | No | YES | NO | NO | Yes | No | YES | Critically Low quality |
| **Loman,2018** | YES | No | Yes | Partial yes | YES | YES | Partial Yes | YES | YES | No | YES | NO | NO | Yes | Yes | YES | Low quality |
| **Liu,2019** | YES | No | Yes | Partial yes | YES | YES | Partial Yes | No | YES | No | YES | Yes | Yes | Yes | No | YES | Critically Low quality |
| **Yang,2021** | YES | No | Yes | Partial yes | YES | YES | Partial Yes | YES | YES | No | YES | Yes | Yes | Yes | No | YES | Critically Low quality |
| **Xio,2019** | YES | No | Yes | Yes | YES | YES | Partial Yes | No | YES | No | YES | NO | NO | Yes | Yes | YES | Critically Low quality |
| **Lavekar,2017** | YES | No | Yes | Partial yes | YES | YES | Partial Yes | No | YES | No | YES | Yes | Yes | Yes | No | YES | Critically Low quality |
| **Li,2022** | YES | YES | Yes | Partial yes | YES | YES | Partial Yes | YES | YES | No | YES | NO | NO | Yes | Yes | YES | Low quality |
| **Huang,2022** | YES | No | Yes | Partial yes | YES | YES | Partial Yes | Partial Yes | YES | No | YES | Yes | Yes | Yes | Yes | YES | Low quality |
| **Koutnikova, 2019** | YES | YES | Yes | Partial yes | YES | YES | Partial Yes | YES | YES | No | YES | Yes | Yes | Yes | Yes | YES | High quality |
| **Gkiourtzis,2022** | YES | YES | Yes | Partial yes | YES | YES | Yes | Partial Yes | YES | No | YES | Yes | Yes | Yes | Yes | YES | High quality |

*Q1:* ***Did the research questions and inclusion criteria for the review include the components of PICO? Q2: Did the report of the review contain an explicit statement that the review methods were established prior to the conduct of the review and did the report justify any significant deviations from the protocol? Q3: Did the review authors explain their selection of the study designs for inclusion in the review?*** *Q4:* ***Did the review authors use a comprehensive literature search strategy?*** *Q5: Did the review authors perform study selection in duplicate? Q6: Did the review authors perform data extraction in duplicate?* ***Q7: Did the review authors provide a list of excluded studies and justify the exclusions?*** *Q****8: Did the review authors describe the included studies in adequate detail?*** *Q****9: Did the review authors use a satisfactory technique for assessing the risk of bias (RoB) in individual studies that were included in the review?*** *Q****10: Did the review authors report on the sources of funding for the studies included in the review?*** *Q****11: If meta-analysis was performed did the review authors use appropriate methods for statistical combination of results?*** *Q****12: If meta-analysis was performed, did the review authors assess the potential impact of RoB in individual studies on the results of the meta-analysis or other evidence synthesis?*** *Q****13: Did the review authors account for RoB in individual studies when interpreting/ discussing the results of the review?*** *Q14: Did the review authors provide a satisfactory explanation for, and discussion of, any heterogeneity observed in the results of the review?****Q15: If they performed quantitative synthesis did the review authors carry out an adequate investigation of publication bias (small study bias) and discuss its likely impact on the results of the review?*** *Q****16: Did the review authors report any potential sources of conflict of interest, including any funding they received for conducting the review?***
